# Temporal analysis of hippocampal CA3 gene coexpression networks in a rat model of febrile seizures

**DOI:** 10.1242/dmm.029074

**Published:** 2018-01-01

**Authors:** Hatylas Azevedo, Nathália Amato Khaled, Paula Santos, Fernanda Bernardi Bertonha, Carlos Alberto Moreira-Filho

**Affiliations:** Department of Pediatrics, Faculdade de Medicina, University of São Paulo (FMUSP), São Paulo, 05403-000, Brazil

**Keywords:** Febrile seizures, Coexpression networks, Epilepsy, Microarray, Gene expression profile, Network analysis

## Abstract

Complex febrile seizures during infancy constitute an important risk factor for development of epilepsy. However, little is known about the alterations induced by febrile seizures that make the brain susceptible to epileptic activity. In this context, the use of animal models of hyperthermic seizures (HS) could allow the temporal analysis of brain molecular changes that arise after febrile seizures. Here, we investigated temporal changes in hippocampal gene coexpression networks during the development of rats submitted to HS. Total RNA samples were obtained from the ventral hippocampal CA3 region at four time points after HS at postnatal day (P) 11 and later used for gene expression profiling. Temporal endpoints were selected for investigating the acute (P12), latent (P30 and P60) and chronic (P120) stages of the HS model. A weighted gene coexpression network analysis was used to characterize modules of coexpressed genes, as these modules might contain genes with similar functions. The transcriptome analysis pipeline consisted of building gene coexpression networks, identifying network modules and hubs, performing gene-trait correlations and examining changes in module connectivity. Modules were functionally enriched to identify functions associated with HS. Our data showed that HS induce changes in developmental, cell adhesion and immune pathways, such as Wnt, Hippo, Notch, Jak-Stat and Mapk. Interestingly, modules involved in cell adhesion, neuronal differentiation and synaptic transmission were activated as early as 1 day after HS. These results suggest that HS trigger transcriptional alterations that could lead to persistent neurogenesis, tissue remodeling and inflammation in the CA3 hippocampus, making the brain prone to epileptic activity.

## Abstract (precis)

**Summary:** We carried out a temporal analysis of hippocampal gene coexpression networks to identify relevant genes in a rat model of hyperthermic seizures. These genes were mostly related to immune response, cell adhesion and neurogenesis.

## INTRODUCTION

Mesial temporal lobe epilepsy (MTLE) is the most common cause of drug-resistant epilepsy (Engel, 2001). Surgical treatment has proved to be successful for pharmacoresistant MTLE, but <1% of the patients are referred to surgery, and usually too late for prevention of psychological disabilities (Engel, 2011). Approximately 40% of MTLE patients have a history of febrile seizures (FS) (Chungath and Shorvon, 2008). Notably, MTLE patients with a history of complex FS (MTLE-FS) exhibit greater hippocampal granule cell loss than patients without such antecedent (Alegro et al., 2012). These patients can also experience increased cognitive deficits, because poor memory acquisition is correlated with granule cell loss in temporal lobe epilepsy (Pauli et al., 2006). Moreover, the analysis of hippocampal CA3 explants obtained after surgery has revealed distinct transcriptional signatures between patients with MTLE-FS and those with no history of complex FS (Bando et al., 2013).

Complex FS are defined as those lasting >15 min, occurring more than once over a 24 h period, or associated with brain infection (French, 2012). They are particularly relevant because children experiencing them exhibit acute hippocampal injury and have abnormalities in hippocampal development (Shinnar et al., 2012). In addition, ∼7% of the children undergoing complex FS will develop epilepsy later in life (Vestergaard et al., 2007).

Animal models have been used to investigate the mechanisms underlying complex FS in humans, because they allow the temporal analysis of biological processes activated after the initial seizures. In these models, immature animals are subjected to episodes of hyperthermia that promote hyperexcitability of the limbic system and lead to prolonged seizures. These models have already been used to characterize long-lasting histological, behavioral and electrophysiological changes in the brain after FS (Dubé et al., 2012). They reproduce several features of human epilepsy with a history of febrile seizures, such as age dependence, hyperthermia, duration of seizures and lack of immediate morbidity (Bender et al., 2004). The synaptic reorganization of dentate granule cells is also observed in both the model and in human epilepsy, and is a histological feature that is correlated with enhanced hippocampal excitability (Bender et al., 2003). However, there is still a knowledge gap concerning the persistent molecular changes in hippocampal neurons that can lead to MTLE after complex FS (Dubé et al., 2012).

MTLE is considered to be multifactorial, with environmental factors and genetic background contributing to the final outcome (Nakayama, 2009). Therefore, exploration of the mechanisms underlying MTLE-FS requires research focused not only on the examination of individual genes, but also on the understanding of the interplay between the genome and the FS insults. To this end, the use of a systems biology approach might reveal how clusters of genes operate at a network level and contribute to the molecular mechanisms of complex brain disorders (Gaiteri et al., 2014). More specifically, this analysis could provide further clarification of the molecular pathways involved in increased brain excitability after FS.

Here, we investigated temporal alterations in hippocampal gene coexpression networks in rats submitted to hyperthermia-induced seizures (HS). We sought to obtain further insights into the hippocampal molecular changes elicited by HS that might contribute to chronic epilepsy. The ventral region of the rat hippocampus was selected for transcriptional profiling because hippocampal hyperexcitability can occur preferentially in the ventral CA3 circuitry (Wu et al., 2005). Moreover, the rat ventral hippocampus is homologous to the human anterior hippocampus, which is the region related to histological changes in patients with MTLE (Toyoda et al., 2013). Our rationale was to investigate transcriptional pathways related to epileptogenesis to identify potential targets for therapeutic interventions aimed at disrupting the onset or progression of MTLE-FS.

## RESULTS

### Induction of HS in immature rats

The post-induction behavioral alterations were analyzed after the animals were subjected to a hyperthermic insult. Behavioral seizures were classified according to the Racine scale. Approximately 60% of the animals exhibited seizures after hyperthermia. Among these animals, 45% showed head nodding (stage 2), 10% exhibited forelimb clonus (stage 3), 16% displayed forelimb clonus with rearing (stage 4), and the remaining 29% showed forelimb clonus with rearing and falling (stage 5).

### Differentially expressed genes in the CA3 hippocampus of rats that displayed HS

Differentially expressed (DE) genes were statistically determined at four post-HS stages to determine individual gene expression changes between HS and control (CTRL) groups. The main DE genes in terms of fold changes and the enriched functions for each time interval are displayed in Tables 1-3. Microarray results were validated using qPCR experiments for selected genes (Fig. S1). These genes were selected based on their differential expression at specific time intervals and relevance for epileptic processes.

**Table 1.**
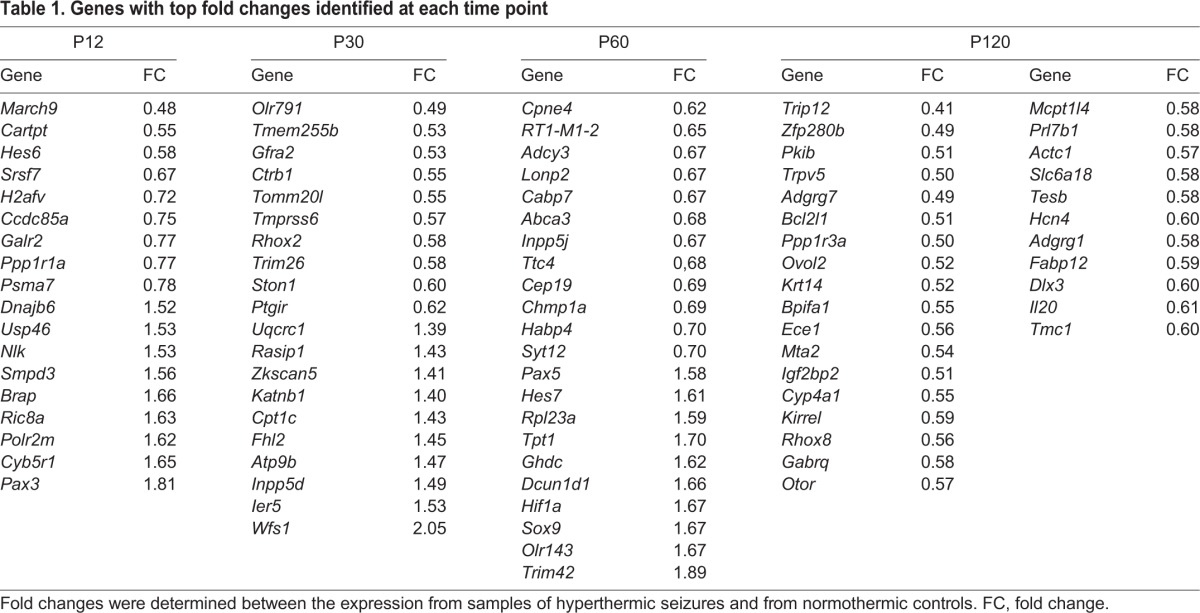
**Genes with top fold changes identified at each time point**

**Table 2.**
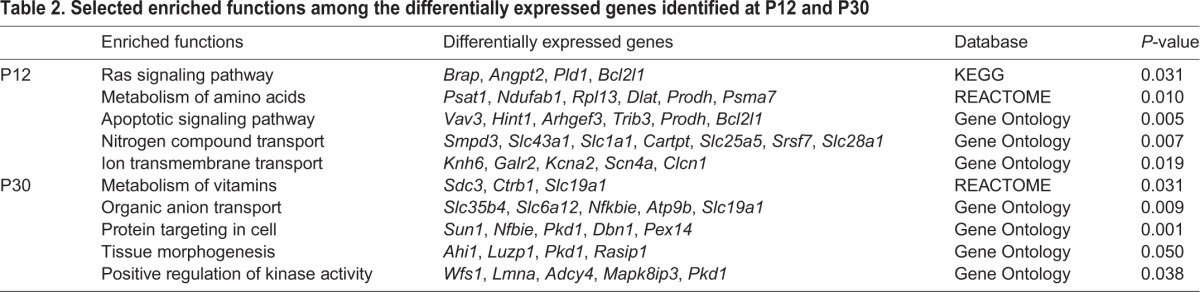
**Selected enriched functions among the differentially expressed genes identified at P12 and P30**

**Table 3.**
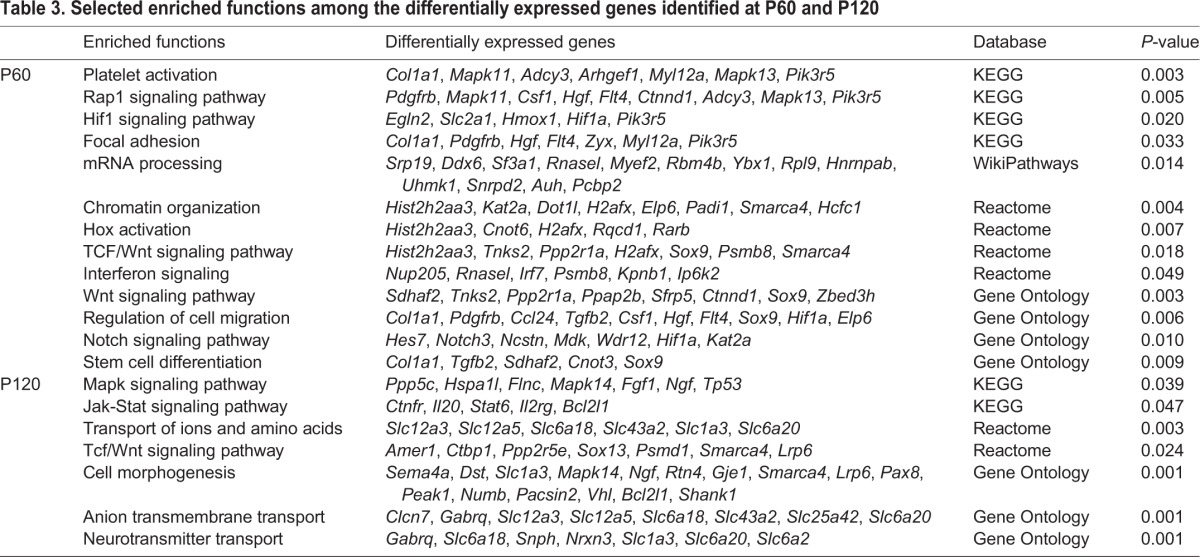
**Selected enriched functions among the differentially expressed genes identified at P60 and P120**

#### Postnatal day 12 analysis

Eighty-nine DE genes (80 up- and nine downregulated) were observed between the experimental groups at postnatal day (P) 12. These genes were found to be associated with the Ras pathway, amino acid metabolism, apoptosis and ion transmembrane transport. Among these genes, the downregulated gene *Hes6* participates in neuronal differentiation (Bae et al., 2000) whereas the upregulated gene *Nlk* codes for a kinase that contributes to cell proliferation and differentiation during development of the nervous system (Ishitani and Ishitani, 2013).

#### P30 analysis

The 83 DE genes (56 up- and 27 downregulated) at P30 were related to organic anion transport, tissue morphogenesis and positive regulation of kinase activity. Interestingly, the protein encoded by the downregulated gene *Gfra2* influences the severity of kindling-evoked seizures in mice (Nanobashvili et al., 2003).

#### P60 analysis

The 263 DE genes (162 up- and 101 downregulated) at P60 were related to Wnt, Rap1, Notch, Hif and interferon signaling pathways, platelet activation, focal adhesion, mRNA processing, chromatin organization, regulation of cell migration and cell differentiation. Among these genes, the upregulated genes *Sox9* (Guo et al., 2012) and *Tpt1* (Johansson and Simonsson, 2010) play a role in the self-renewal of stem cells.

#### P120 analysis

The 341 downregulated genes were mostly associated with Mapk, Jak-Stat and Wnt pathways, transport of ions and amino acids, cell morphogenesis and transport of neurotransmitters. In particular, the downregulated genes *Rhox8* (Artegiani et al., 2015) and *Mta2* (MuhChyi et al., 2013) are involved in neurogenesis.

### Modules and hubs related to hyperthermic seizures at each time interval

We analyzed coexpression patterns in CA3 hippocampal transcriptome data to reveal modules of coexpressed genes and their relationship with a seizure-related state. We constructed two separate networks for each time interval using, respectively, the HS and the CTRL samples. This analysis allowed the comparison of module preservation and connectivity between CTRL and HS networks.

**Fig. 1.**
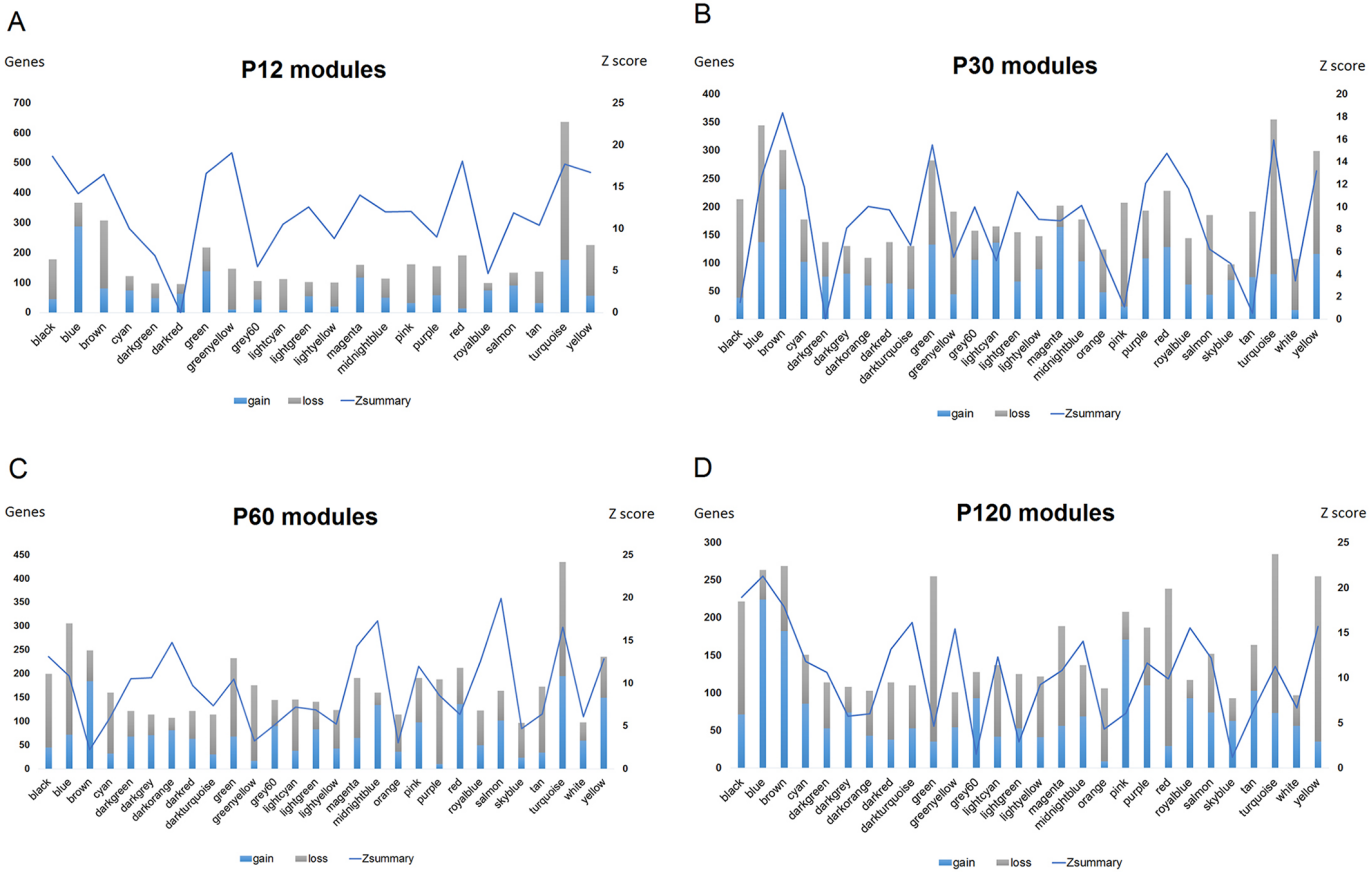
**Analyses of module preservation and intramodular connectivity changes between HS and CTRL networks.** Genes were ranked according to their intramodular connectivity, and changes in ranking positions were determined between networks for identifying nodes and modules associated with gain or loss of connectivity. The summary statistic Zsummary is used to assess preservation of module density and connectivity between the networks. Zsummary<2 denotes no preservation, 2<Zsummary<10 indicates weak to moderate evidence of preservation, and Zsummary>10 suggests strong module preservation.

Nodes were rank ordered by their intramodular connectivity and compared between the HS and CTRL networks. This analysis allowed the evaluation of module preservation between the networks and the identification of modules that gain connectivity in the HS network (Fig. 1). Modules that show increased connectivity are considered to be related to gain of function in the HS group. The hubs and main functions enriched by the genes in these modules are shown in Tables S1-S4.

#### P12 analysis

The top modules associated with gain of connectivity in the P12 comparison were blue, green, magenta and salmon, whereas the top modules that lost connectivity at P12 were brown, red and turquoise (Fig. 1A). Modules less preserved between the CTRL and HS networks at P12 were darkred, royalblue, grey60 and darkgreen. Among these modules, the blue module showed the highest number of genes with gain of connectivity. This module exhibited enriched functions related to apoptosis, regulation of cell adhesion and migration, cellular response to stress and axonogenesis. The hub *Bcar1* in this module codes for an adaptor protein relevant to cell migration and axon elongation (Huang et al., 2006), whereas the hub *Entpd2* is a gene that controls progenitor cell proliferation in brain neurogenic niches (Gampe et al., 2015).

The turquoise module also encompassed many genes showing gain of connectivity. This module was associated with apoptosis, tight junctions, synaptic transmission, neuron differentiation, immune-related pathways, the Hippo pathway and the Wnt pathway. The hubs *Rtn2* and *Rtn3* in the module turquoise are part of the reticulon protein family that plays a role in neuronal sprouting. Indeed, mice overexpressing *Rtn3* were described to develop neuritic abnormalities (Hu et al., 2007). The darkred module was the less preserved module at P12. This module was related to pathways linked to neurotrophin, Fgfr, Hif1 and Wnt, apoptosis, activation of NMDA receptors, cell differentiation and axonogenesis. The hub *Nefl* in this module is a marker of differentiated neurons, whereas the hub *Ssbp3* encodes a protein that induces the differentiation of embryonic stem cells into trophoblast-like cells (Liu et al., 2016).

#### P30 analysis

The top modules associated with gain of connectivity at P30 were brown, green, lightcyan, magenta and red, whereas modules that lost connectivity at P30 were black, blue, greenyellow, pink, turquoise and yellow (Fig. 1B). Modules less preserved between the CTRL and HS networks at P30 were darkred, turquoise, pink, darkturquoise and green. Among the modules in the P30 network, the brown module showed the highest number of genes with gain of connectivity. This module showed enriched functions related to cellular metabolism, protein degradation pathways, cell differentiation, apoptosis and synaptic transmission. The hub *Osm* in this module encodes oncostatin M, which inhibits the proliferation of neural precursor cells (Beatus et al., 2011).

The magenta module also showed many genes with connectivity gain. These genes were associated with the phospholipase D pathway, actin cytoskeleton organization, regulation of cell adhesion and chemotaxis, positive regulation of neurogenesis and regulation of stem cell maintenance and differentiation. For example, the hub gene *RalA* (Ras-like small GTPase) is involved in cell polarization during neuronal development (Das et al., 2014). The darkgreen module was less conserved in the P30 network. This module was related to axon guidance, long-term potentiation, apoptosis, the Bdnf pathway, the Il1 pathway, cell adhesion, EPH-Ephrin signaling, innate immune pathways and Rho GTPase effectors. The hubs *Pif1*, *Chac1* and *Noxa1* in this module are associated with cell viability and oxidative stress, whereas the hub *Epha10* participates of the EPH-Ephrin signaling, which is involved in axon guidance and is activated in the hippocampus of pilocarpine-treated mice (Xia et al., 2013).

#### P60 analysis

The top modules associated with gain of connectivity at P60 were brown, midnightblue, pink, red, salmon, turquoise and yellow, whereas the top modules that lost connectivity at P60 were black, blue, green and magenta (Fig. 1C). Modules less preserved between the CTRL and HS networks at P60 were grey60, skyblue, greenyellow, orange and brown. The brown and turquoise modules exhibited many genes with gain of connectivity. The brown module was also the less preserved module in the P60 network. Genes in the brown module were related to focal adhesion, stem cell pluripotency, lymphocyte proliferation, cell migration and the development of neuron projections. Among the hubs in this module, *Actn1*, *Fkbp8* and *Acap3* are involved in neurite extension*.* In particular, hippocampal neurons have shown abolished neurite outgrowth after *Acap3* knockdown (Miura et al., 2016). Other hubs were *Cacnb3*, a voltage-activated calcium channel, and the immune-related genes *Cnrip* and *Il16*.

The main enriched functions for the turquoise module were calcium and potassium transport, axon guidance and neuron migration, developmental pathways, such as Wnt and Robo, synaptic transmission and inflammatory regulation of TRP channels. Among the hubs in the turquoise module, the hub gene *Scx* (scleraxis) encodes a transcription factor that is important to determination of the fate of stem cells. The hub *Mx1* codes for a protein expressed in oligodendrocytes and the hub *Ednra* encodes an endothelin receptor that acts as an axonal guidance cue for sympathetic neurons (Makita et al., 2008). Finally, the greenyellow module was also little preserved at P60. This module was related to the Wnt pathway, the p53 pathway, axonal guidance, neuron cell morphogenesis, protein ubiquitylation and synaptic transmission. Among the hubs in this module, the gene *Ptprt* codes for a tyrosine kinase that regulates synaptic formation and neuronal development (Lee, 2015). Other relevant hubs were *Pomgnt1*, which encodes an enzyme that performs protein glycan modification during brain development (Dwyer et al., 2015), and *Taok2*, whose encoded kinase is essential for dendrite morphogenesis (de Anda et al., 2012).

#### P120 analysis

The top modules associated with gain of connectivity at P120 were blue, brown, grey60, pink, purple, royalblue and tan, whereas the top modules that lost connectivity at P120 were black, green, magenta, red, turquoise and yellow (Fig. 1D). Modules less preserved between the CTRL and HS networks at P120 were green, grey60, lightgreen, orange and skyblue. Genes in the blue module were associated with the Mapk pathway, extracellular matrix organization, synaptic transmission, apoptosis, cell differentiation, regulation of neurogenesis, neuron projection guidance and response to oxidative stress. Among the hub genes in this module, *Ndufb3* and *Ndufs5* participate in the process of oxidative phosphorylation, *Npdc1* is a regulator of neuronal proliferation and differentiation, and *Shank3* modulates NMDA receptor levels at axon terminals (Halbedl et al., 2016). The brown module was related to synaptic transmission, focal adhesion, regulation of the actin cytoskeleton, axon guidance, signaling by Wnt, signaling by Robo and neuron differentiation. Among the hubs in this module, *Gria1* codes for a glutamate AMPA receptor and *Robo2* is part of the Robo pathway that regulates axon guidance.

The grey60 and skyblue modules were the less preserved in the P120 analysis. The grey60 module was associated with immune functions such as Fc gamma R-mediated phagocytosis, lymphocyte proliferation and activation, regulation of nuclear factor-κB signaling, cell projection organization and cell morphogenesis. Among the hubs in this module, *Gatad2a* is part of the chromatin-remodeling complex NuRD, which regulates dendrite pruning and neuronal connectivity (Yamada et al., 2014). In parallel, the skyblue module was related to the tumor necrosis factor-α–nuclear factor-κB pathway, hyaluronan metabolism, the Wnt pathway, neuron differentiation and cell migration. The hub *Ska3*, for example, encodes a spindle checkpoint protein that promotes neurite outgrowth (Tong et al., 2013). Moreover, *Hyal2* and *Hmmr* participate of hyaluronan metabolism, which is relevant for cell motility, whereas *Avl9* is also involved in cell migration.

### Modules and genes associated with seizure susceptibility or resistance time intervals

**Fig. 2.**
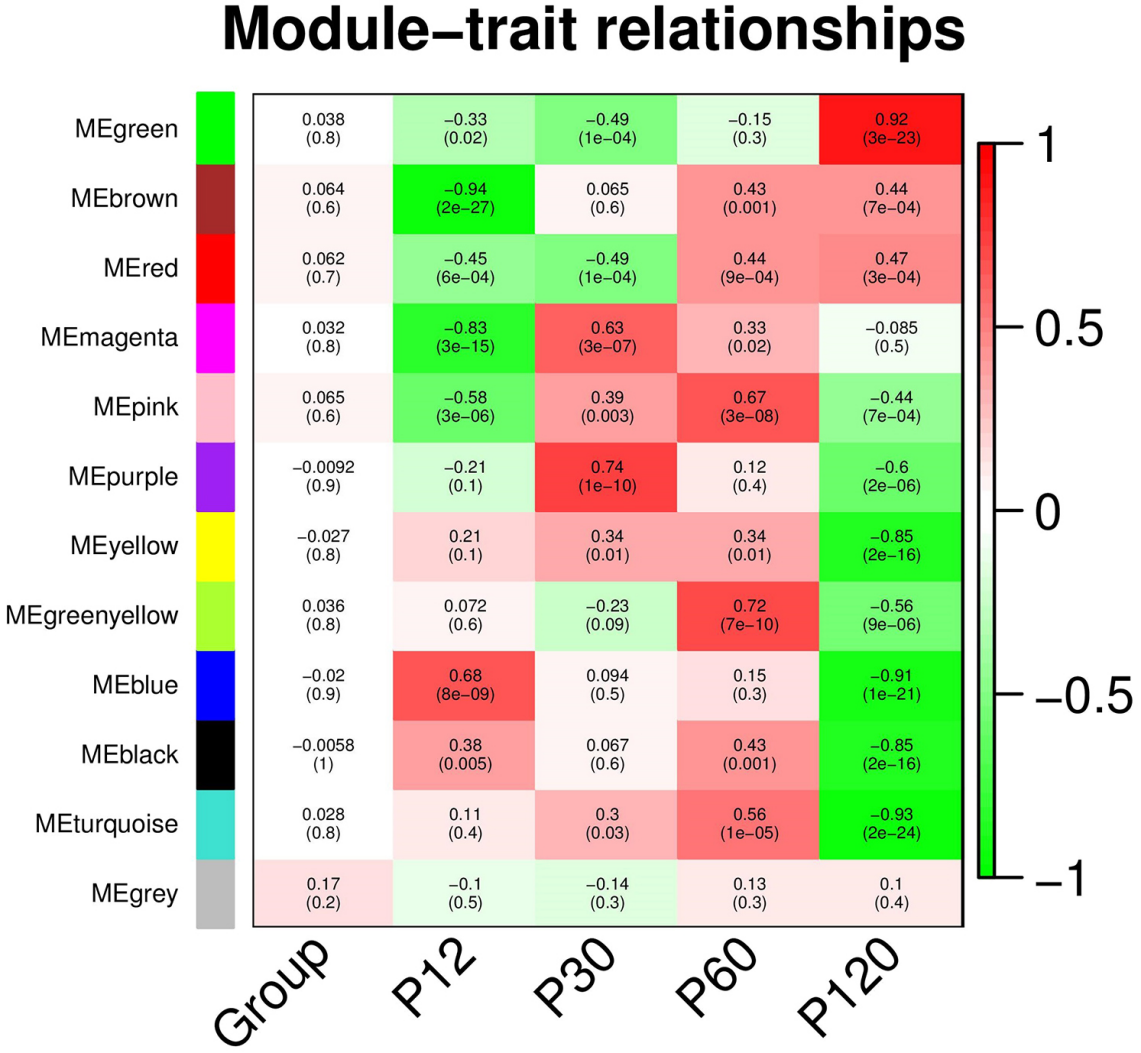
**Module-trait relationships from the WGCNA analysis, in which networks were built using all microarray samples.** Module names are displayed on the left, and the correlation coefficients to the hyperthermic seizures (HS) group are shown at the top of each row. The corresponding *P*-values for each module are displayed at the bottom of each row within parentheses. The rows are colored based on the correlation of the module with the HS group: red for positive and green for negative correlation.

A single coexpression network was also constructed using the gene expression data from all samples obtained in this study. The modules obtained in this network were then correlated with each time interval for disclosing modules linked to specific time intervals (Fig. 2). This analysis allowed the identification of modules correlated with time intervals involved in seizure resistance (P12 and P30) and susceptibility (P60 and P120). For example, the blue module was positively correlated with P12 and the magenta and purple modules were positively correlated with P30. The pink, green, yellow and turquoise modules were in turn mainly related to P60. Finally, the green module was positively correlated with P120, and the modules brown and red were correlated with P60 and P120.

**Fig. 3.**
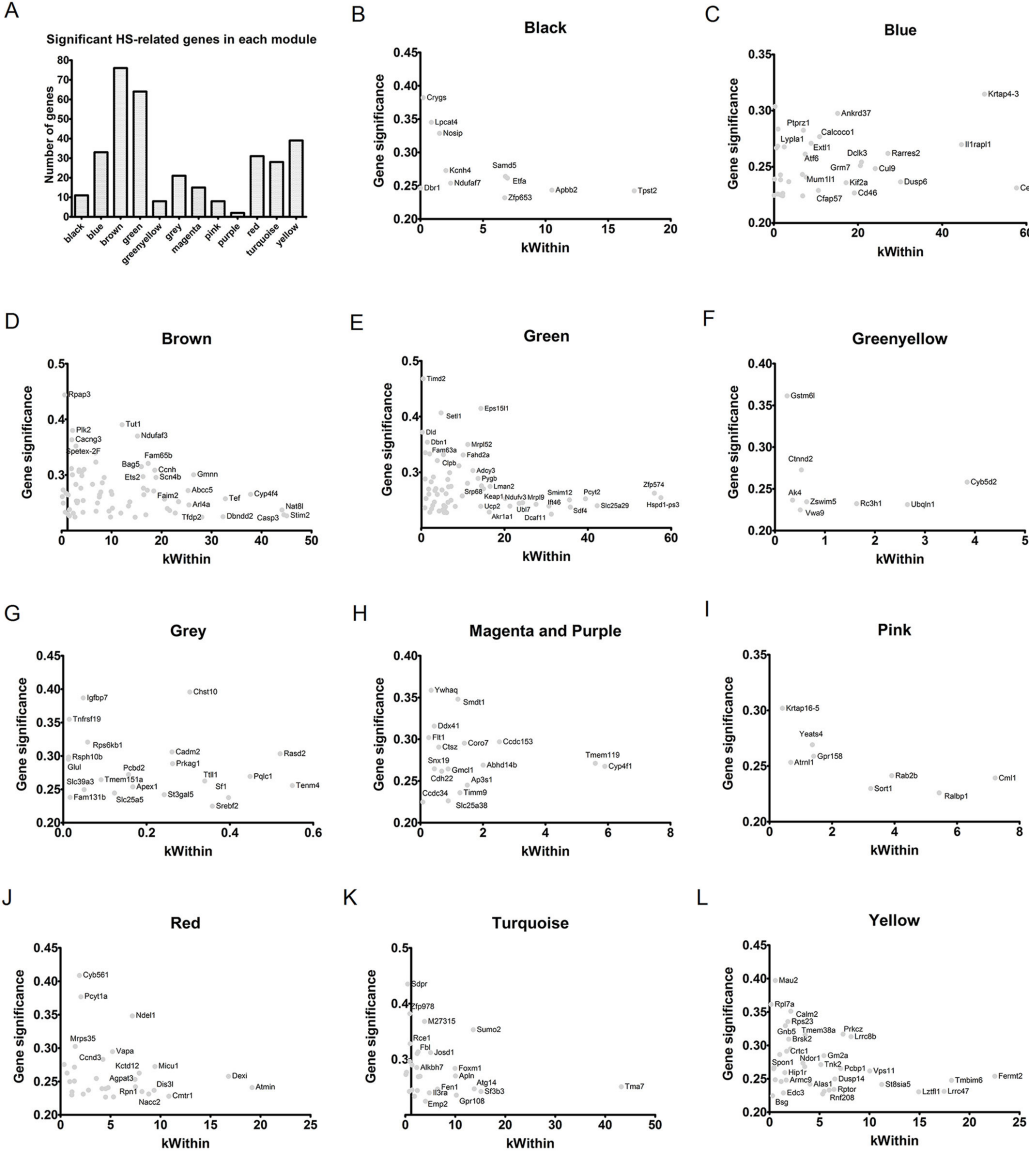
**Significant genes correlated with the HS group in each module, based on the analysis depicted in Fig.** **2.** Specific genes in each module were significantly associated with the HS group. (A) The number of genes in each module that displayed a *P*-value for gene significance (GS) <0.1. (B-L) Scatter plots constructed using the K_within_ (*x*-axis) and GS (*y*-axis) values for each gene in their corresponding modules.

Although none of the above-mentioned modules was significantly correlated with the HS group, specific genes in these modules were significantly associated with the HS group. After filtering the genes with the *P*-value for gene significance (GS) <0.1 (Fig. 3A), we built scatter plots using the K_within_ (intramodular connectivity) and GS values for each module (Fig. 3B-L). These plots supported the selection of some genes for further detailing in each module.

#### P12 analysis

In the blue module, the genes *Ptprz1*, *Lypla1*, *Mum1l1*, *Grm7*, *Atf6* and *Il1rapl1* showed relative high K_within_ and GS values. *Lypla1* was the highest connected node in this module. *Lypla1* codes for a thioesterase that regulates protein palmitoylation during dendritic spine morphogenesis (Siegel et al., 2009). In addition, the hubs *Grm7* (glutamate receptor, metabotropic 7) and *Il1rapl1* (Interleukin-1 receptor accessory protein-like 1, which regulates the formation of glutamatergic synapses; Hayashi et al., 2013), revealed the transcriptional regulation of the glutamatergic signaling at P12.

#### P30 analysis

For the modules associated with P30, the nodes *Smdt1*, *Ccdc153*, *Cyp4f1*, *Tmem119* (module magenta) and *Gmcl1* and *Ddx41* (purple module) were the most important genes related to the HS group. *Tmem119* is a marker of resident microglia in human brain (Satoh et al., 2016), whereas *Cyp4f1* expression is increased in hippocampal astrocytes of rats subjected to brain injury (Wang et al., 2008).

#### P60 analysis

The pink, greenyellow and turquoise modules in turn were mainly related to P60. The main hubs associated with the HS group were *Cyb5d2*, *Ubqln1*, *Rc3h1* (greenyellow), *Cml1*, *Ralbp1* and *Rab2b* (pink), and *Tma7*, *Sumo2*, *Apln* and *Foxm1* (turquoise). Among these genes, *Ubqln1* (Zhang et al., 2015) and *Ralbp1* (Bae et al., 2013) regulate seizure threshold via the GABAergic signaling. In parallel, *Rab2b* (Ayala et al., 1990) and *Foxm1* (Ueno et al., 2008) play a role in neuronal differentiation. Finally, the genes *Cyb5d2*, *Sumo2* (Datwyler et al., 2011) and *Apln* (Zhang et al., 2011) participate in injury protection mechanisms.

#### P120 analysis

The brown and red modules were also correlated with the late stages of the experimental model (P60 and P120). Several of the hubs and genes with top GS values in the brown module are closely related to epilepsy. *Stim2* encodes a calcium sensor protein that modulates neuronal activity in a model of chronic epilepsy (Steinbeck et al., 2011). *Plk2* is a gene required for plasticity of hippocampal neurons during epileptiform activity (Seeburg and Sheng, 2008), whereas *Cacng3* encodes a calcium channel associated with childhood absence epilepsy (Everett et al., 2007). For the red module, some of the relevant genes in terms of K_within_ or GS were *Atmin*, which is protective against oxidative stress in the aging brain (Kanu et al., 2010), and *Ndel1*, which regulates neuronal migration (Sasaki et al., 2005). Finally, the green module (linked to P120) exhibited many genes significantly correlated with the HS group. Among the hubs in this module, *Slc25a9*, *Ucp2*, *Ndufv3*, *Mrpl9* and *Hspd1* are all located in mitochondria.

### Frequent functions related to hyperthermic seizures at different time points

**Fig. 4.**
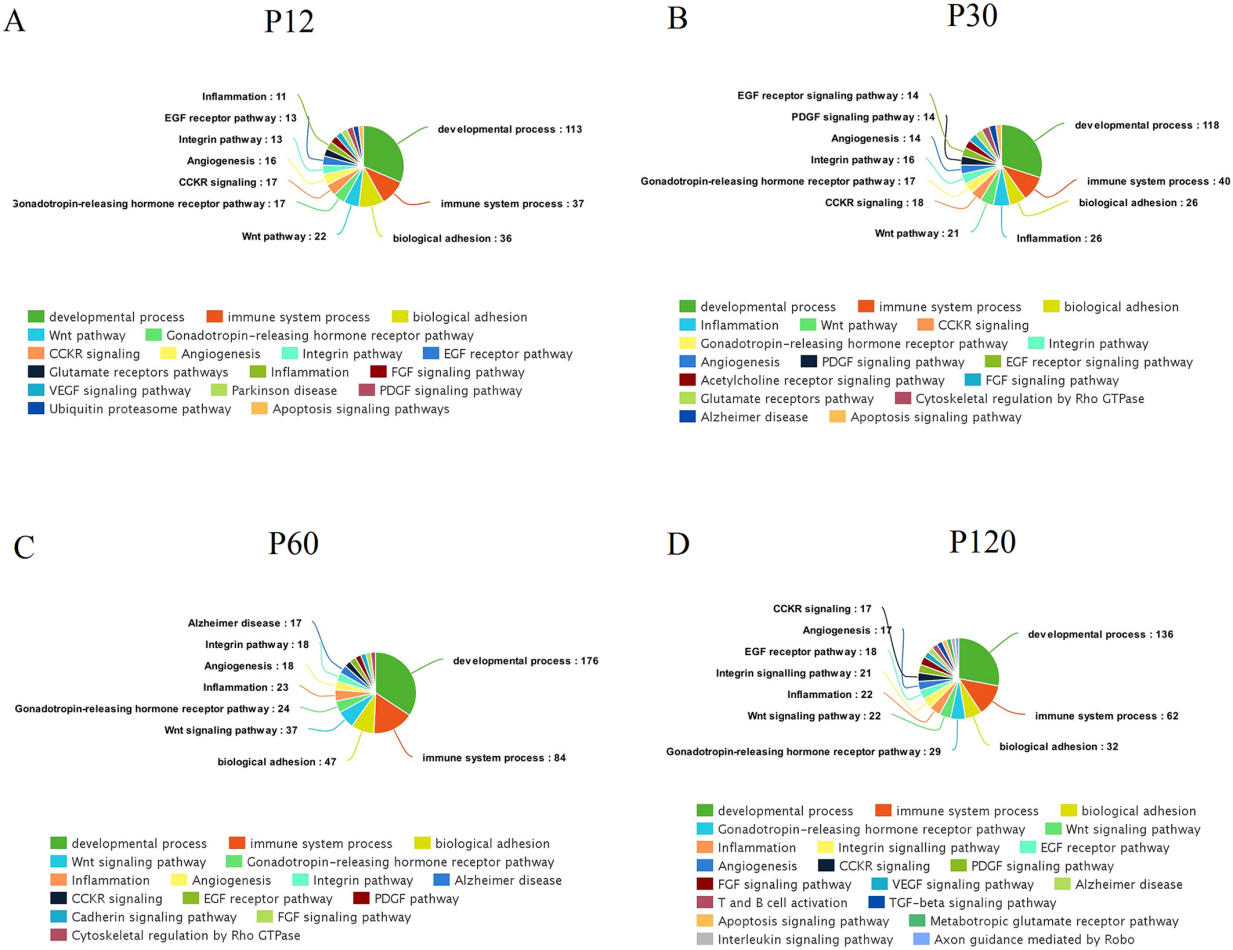
**Pie charts showing the percentage of genes for selected enriched biological functions and pathways at different time points after hyperthermic seizures.** (A-D) The functions identified in the modules that gain connectivity in the HS networks. Networks were constructed using the HS and CTRL samples separately, to allow comparison between HS and CTRL networks at each time point.

We also constructed pie charts to visualize the functions that exhibit the highest number of genes in the specific modules related to hyperthermic seizures at each time interval. The pie charts showing the number of genes in each function are depicted in Fig. 4. Fig. 4A-D exhibits the analysis performed in the modules from the networks built using the HS and CTRL samples separately for each time interval. This analysis showed that modules related to hyperthermic seizures were mostly associated with developmental processes, immune system processes and biological adhesion.

## DISCUSSION

In this study, we investigated the molecular pathways underlying the process by which neonatal FS could contribute to seizure recurrence at a later age, using a rat model of HS. The temporal analysis of hippocampal gene coexpression networks enabled the identification of coexpression modules and hub genes relevant to the acute, latent and chronic phases of the animal model. In general, hub genes and HS-related modules were mostly related to functions involved in the immune response, cell adhesion and neurogenesis.

Inflammation-related functions were enriched by relevant modules during the acute and advanced stages of the experimental model. Previous experiments have disclosed mechanisms by which inflammation mediates epileptogenesis, leading to the development of MTLE after complex FS (Choy et al., 2014). Moreover, inflammatory markers are increased in the hippocampus and are correlated with the development of epilepsy in the rat model of HS (Patterson et al., 2015).

Cell adhesion functions were also frequently observed in relevant modules from hippocampal gene coexpression networks of rats that displayed HS. Notably, cell adhesion molecules participate in many biological functions relevant for the development of epilepsy, such as inflammatory processes, synaptic plasticity, signal transduction, neuronal sprouting and cell migration (Engel et al., 2008). In fact, experimental FS trigger a transcriptional response that leads to tissue structural remodeling in the hippocampus during the acute and latent phases of the HS model in mice (Jongbloets et al., 2015).

Genes and modules associated with neurogenesis were regulated in all evaluated time intervals. Neurogenesis in the hippocampal dentate gyrus was previously characterized after neonatal FS (Scott and Holmes, 2012). These cells are generated in the subgranular zone and migrate to the dentate granular cell layer. The ectopic granule cells send their axons and form excitatory connections with CA3, leading to enhanced hippocampal excitability after FS (Scott and Holmes, 2012). Nevertheless, neurogenesis is still poorly understood in the CA3 region. Hippocampal neurogenesis was already shown to occur in CA3 after kainic acid administration in neonatal rats at P7. In these animals, the number of bromodeoxyuridine-positive cells increased in CA3 at P40 and P60, suggesting a late stimulation of neurogenesis by kainic acid in neonatal rats (Dong et al., 2003). Neural stem cells from CA3 are capable of differentiating *in vitro* into neurons, astrocytes and oligodendrocytes. Interestingly, postnatal age influences the extent to which progenitor cells give rise to differentiated cells in CA3. It is observed that neuronal differentiation decreases, whereas oligodendrocyte differentiation increases with age (Shetty and Hattiangady, 2013).

Biological functions activated at P12 revealed the response to brain injury immediately after HS, such as amino acid metabolism, apoptosis, immune response, response to cellular stress and ion transmembrane transport. Interestingly, many functions related to neuronal differentiation, axonogenesis and the regulation of cell migration were already activated 1 day after the episode of hyperthermia. In fact, recent data have suggested that neurogenesis is induced by acute seizures or precipitating insults, whereas the capacity for neuronal recruitment and proliferation substantially decreases in the chronic phase of epilepsy (Huang et al., 2015).

The regulation of several genes from the Hippo and Wnt pathways at P12 suggests that seizure-induced injury might recapitulate pathways related to development and homeostasis in the brain. These two pathways are interconnected and regulate one another to promote a proper tissue response (Konsavage and Yochum, 2013). Moreover, the fact that the Wnt pathway modulates neuroimmune interactions after injury places this pathway as an intersecting player between inflammation and neurogenesis (Marchetti and Pluchino, 2013).

Modules involved in glycolysis and gluconeogenesis were related to the HS group at P30, a time point linked to a seizure-resistant phenotype. Previous studies have shown that glycolysis increases during seizures, generating lactic acid and providing energy during seizures. However, lactic acid reduces tissue pH and generates metabolic acidosis, which ultimately terminates seizures (Yang et al., 2013). Therefore, one of the mechanisms underlying seizure tolerance at P30 might be linked to the metabolic acidosis in the brain.

Relevant modules at P30 were also involved in neurogenesis, such as cell differentiation, axon guidance, stem cell differentiation and regulation of cell adhesion. Indeed, genes linked to the phospholipase D and EPH-Ephrin pathways were associated with gain of connectivity at P30. Phospholipase D promotes the release of tissue plasminogen activator, initiating a proteolytic cascade of extracellular matrix components and facilitating neurite outgrowth (Zhang et al., 2005). In parallel, EPH-Ephrin signaling is increased in epileptic hippocampus, resulting in axonal sprouting and epileptogenesis (Xu et al., 2003). Modules linked to innate immune pathways were also identified at P30, together with hubs related to microglia and astrocyte markers. This observation highlights that proconvulsant events can activate microglia and astrocytes to release inflammatory mediators, initiating a cascade of events that might affect neuronal excitability. The Delta-Notch pathway was also an enriched function in relevant modules at P30. This pathway induces proliferation during neurogenesis but also promotes neuronal excitation when activated after seizures (Sha et al., 2014).

Modules correlated with the P60 interval were involved in immune functions, chromatin organization, synaptic development, cell migration, the Wnt pathway, focal adhesion, potassium transport, axon guidance and neuron migration. This time interval might be particularly important to epileptogenesis, because epigenetic-related functions were observed at P60, which might account for the long-lasting molecular alterations induced by febrile seizures. In parallel, the expression of focal adhesion genes suggests that the interaction between the extracellular matrix and brain cells is important for the epileptogenic process that generates the hyperexcitable tissue after initial seizures. Indeed, focal adhesion signaling plays a role in hippocampal mossy fiber sprouting in the pentylenetetrazole kindling model (Song et al., 2015). In addition, the gene expression regulation of potassium channels might be related to the kainate seizure susceptibility phenotype observed at this time interval.

Finally, the modules related to the P120 interval were mostly involved in the oxidative stress response, apoptosis, cell differentiation, cell migration, immune pathways and developmental signals, such as Hippo, Polycomb repressive complex 2, Robo and Wnt. The fact that modules associated with oxidative stress and mitochondrial dysfunction were strongly related to P120 indicates that mitochondrial respiration deficits and the resultant oxidative stress play a role in chronic epilepsy (Rowley and Patel, 2013). Interestingly, the transcriptional regulation of genes related to the Polycomb repressive complex 2 (Prc2) indicates that a long-term silencing of genes involved in stem cell pluripotency and differentiation might be regulated by Prc2 at P120. This can also be correlated with the several downregulated genes observed at P120 in this experimental model. Interestingly, it was recently shown that PRC2 silences genes responsible for neurodegeneration in mouse striatal neurons (von Schimmelmann et al., 2016).

Other interesting functions were also enriched in HS-related modules at P30, P60 and P120. The Cck (Cholecystokinin) signaling pathway, for example, is inversely associated with pharmacoresistance in epilepsy, because it controls the activity of hippocampal inhibitory interneurons (Mirza et al., 2011). Another relevant function was the gonadotropin releasing hormone (GnRH) pathway, which was involved in epileptogenesis after enrichment analysis of microarray datasets (Subramanian et al., 2005).

This study has some limitations. The first one is related to the use of mixed cell populations from hippocampal CA3 samples, which does not allow the determination of the roles of specific cell types among the identified transcriptional changes. The other limitation is the inclusion of animals with distinct seizure behaviors in the same experimental group. This approach was taken for reasons of feasibility, because otherwise a much larger number of animals would be necessary, considering the mortality inherent to the protocol, the number of time points and phenotypic variability issues.

### Conclusions

To our knowledge, this is the first study to provide a temporal network analysis of rat CA3 hippocampal gene expression profiles after hyperthermia-induced seizures. The analysis revealed transcriptional modules that might relate to the development of epilepsy after febrile seizures, contributing to the identification of potential targets for the therapeutic treatment of MTLE-FS. Our data suggest that drugs interfering with developmental and immune pathways, such as Wnt, Hippo, Notch, Jak-Stat and Mapk, could constitute the basis of effective therapies for preventing the onset of MTLE after early-life febrile seizures. Further studies using orthogonal methods, such as immunohistochemistry, will help to identify specific hippocampal cell types related to changes observed after hyperthermic seizures.

## MATERIALS AND METHODS

### Animal model of hyperthermic seizures

The experimental model used here is similar to the original model of HS in immature rats described by Baram et al. (1997). Briefly, Wistar rats were allowed to mate during 4 days consecutively in a standard 12 h light-12 h dark cycle. The age of the pups was determined from the day of birth (P0). At P11, animals were subjected to hyperthermia (39.5-42.3°C) in a glass box with incandescent lamps until they reached a body temperature of 39°C for 45 min. The control group consisted of animals that were placed in the glass container but were not exposed to the heat insult. Animals that underwent a 45 min hyperthermic interval were subsequently monitored in euthermic conditions for 1 h. The post-induction interval was video recorded, and behavioral seizures were classified according to the Racine scale, as follows: orofacial automatisms (stage 1); head nodding (stage 2); forelimb clonus (stage 3); forelimb clonus with rearing (stage 4); and forelimb clonus with rearing and falling (stage 5). The Ethics Committee of Faculdade de Medicina, University of São Paulo approved this study under the number 460/13.

### Experimental design

Rat pups in equal male-to-female ratio (*n*=6-8 per group and time interval) were divided into two groups: control (CTRL) and animals that developed seizures after the hyperthermic treatment (HS). Only those animals that exhibited seizures classified as stage 2 or higher in the Racine scale were assigned to the HS group. The selection of the temporal endpoints was based on previous studies showing associations between specific time intervals and seizure susceptibility or resistance after pharmacological seizure induction. For instance, a decrease in the incidence of seizures generated by pentylenetetrazole was observed after 24 h (P12), and after 20 days following the episode of hyperthermia (Gonzalez-Ramirez et al., 2009). Conversely, animals submitted to hyperthermic seizures at P11 are prone to develop seizures at P60 (Zhao et al., 1985) and P90 (Dube et al., 2000) when treated with a subconvulsive dose of kainate. In this experimental model, the majority (90%) of the animals exhibit interictal epileptiform discharges (Dubé et al., 2006), and spontaneous seizures occur in 45% of the animals, starting at ∼3-4 months (P120) of age (Dubé et al., 2010).

### RNA extraction from ventral CA3 hippocampus

Brain tissue samples were collected from the ventral CA3 hippocampus at 1 (P12), 19 (P30), 49 (P60) and 109 (P120) days after hyperthermia-induced seizures. These time intervals were selected for evaluation of the acute (P12), latent (P30 and P60) and chronic (P120) stages of the experimental model. Brain microdissection was performed as previously described (Gorter et al., 2006). Briefly, after decapitation, the temporal lobe and hippocampus were removed by incision at the ventrocaudal part underneath the rhinal fissure, until 5 mm posterior to bregma. Then, the hippocampus was cut into smaller pieces (200-300 µm), and the CA3 region was selected and removed in PBS at 4°C under a dissecting microscope. The CA3a and CA3b regions were included in the analysis, and the dentate gyrus was not sampled. The material obtained from the ventral CA3 region was placed in 0.5 ml Eppendorf tubes containing RNA*later* (Qiagen) for subsequent total RNA extraction. Total RNA was extracted using RNeasy^®^ Mini Kit (Qiagen) and stored at −80°C until use in subsequent experiments. RNA integrity was analyzed in all samples using a Bioanalyzer 2100 (Agilent Technologies, Santa Clara, CA, USA). The analysis performed by the Bioanalyzer platform calculates an RNA integrity parameter (RIN), which is based on the ratio of 28S:18S bands (Schroeder et al., 2006). RIN values range from 10 (intact) to 1 (totally degraded). We only used samples with RIN≥7.

### Oligonucleotide microarray data analysis

Microarray data were obtained as previously described (Correa-Costa et al., 2012). Gene expression experiments were accomplished in the Agilent microarray platform, according to the manufacturer's instructions. Agilent whole rat genome 4X44K v3 oligonucleotide microarrays (G2519F-028 282) were used to examine the transcriptional profiles. The R environment (http://www.r-project.org) was used to analyze the data. The processed signal (gProcessedSignal) generated by software Feature Expression (v9.5.3) was used for further analyses. The mean expression value for each gene was calculated, and the data were log_2_-transformed. Data were processed and normalized by quantile normalization using the Limma package in R (Smyth, 2005). The DE genes were identified using the significance analysis of microarrays algorithm implemented in TMeV (Saeed et al., 2003), with a false discovery rate of 10% (Benjamini, 1995). The microarray data set is available at the NCBI Gene Expression Omnibus, through the accession number GSE84289.

### Gene coexpression network analysis

The network analysis workflow used here is depicted in Fig. S2. We performed an unsigned weighted gene coexpression network analysis (WGCNA) to identify clusters of coexpressed genes, because genes with correlated expression levels can be associated with common regulatory mechanisms. The WGCNA method calculates a matrix of pairwise correlation coefficients from the expression data and transforms it into an adjacency matrix that contains the coexpression values raised to a soft threshold, β. The β value is selected for maximizing the networks' scale-free topology. The analysis was implemented using the WGCNA package in R, which constructs networks by means of maximizing the scale-free topology fit (Langfelder and Horvath, 2008). The soft threshold of 10 was chosen to maximize the scale-free topology index. The resulting *R*^2^ for this function was >0.8 in all generated networks.

The algorithms were applied for finding clusters (modules) of coexpressed genes, summarizing clusters using the module eigengene, identifying hub genes, relating specific modules to experimental groups or to other modules, and comparing module parameters (e.g. module membership) between networks. In the WGCNA package, different colors are arbitrarily assigned to the modules in order to distinguish them. The modules are summarized by the parameter module eigengene (ME), which corresponds to the first principal component of gene expression variation in each module. The biweight mid-correlation coefficient was used to calculate the similarities in expression between all gene pairs. Only the 5000 most variable genes in each time interval were analyzed in order to reduce background noise. The minimum module size was set to 50 genes.

Gene significance was calculated by correlating each gene expression profile with a sample trait. Module significance was calculated using eigengene significance (correlation between sample trait and eigengene) and its corresponding *P*-value for each module. Only genes with a *P*-value ≤0.05 and modules with a *P*-value ≤0.1 were considered significantly correlated with a sample trait. Intramodular hubs were identified as the10 top-ranked genes based on intramodular connectivity in each module. Genes were ranked according to their intramodular connectivity, and changes in ranking positions were determined between networks for identifying nodes and modules associated with gain or loss of connectivity.

Module preservation statistics (Langfelder et al., 2011) were implemented to evaluate the degree of similarity between the same modules assigned in the networks. The permutation Z score was used to assess module density and connectivity. Zsummary<2 denotes no preservation, 2<Zsummary<10 indicates weak to moderate evidence of preservation, and Zsummary>10 suggests strong module preservation (Langfelder et al., 2011). Genes not clustered into any modules were assigned to grey modules in the WGCNA package, i.e. the grey module might contain genes associated with traits that are not part of a WGCNA module. The gold modules consisted of 1000 random genes selected for the module preservation analyses. When the networks were constructed separately for CTRL and HS groups, modules were identified in the CTRL network, and the corresponding genes for each module were kept the same in the HS network in order to allow network comparison.

### Gene lists enrichment analysis

The online software tools EnrichR (Chen et al., 2013) and PANTHER (Thomas et al., 2003) were used to identify functions overrepresented by lists corresponding to DE genes or to modules of coexpressed genes. The databases Gene Ontology, KEGG, WikiPathways, Reactome and Biocarta were used for selecting enriched functions. Only functions displaying a *P*-value ≤0.05 and encompassing at least three enriched genes were considered significant. No background gene set was used for the functional enrichment analysis. Similar functions were grouped together to facilitate the analysis of the functional enrichment results. Pie charts were constructed to visualize enriched functions with the highest number of genes at each time interval using META-CHART (https://www.meta-chart.com/pie#/data).

### Quantitative polymerase chain reaction

Validation of microarray results was performed by qPCR. This procedure was carried out using the SuperScript^®^ III Reverse Transcriptase (Life Technologies, USA) and QuantiFast SYBR Green PCR (Qiagen, USA) kits. Samples were incubated at 95°C for 5 min and subjected to 40 cycles of 95°C for 30 s and 60°C for 30 s. Primers were designed in the Primer 3 software (http://bioinfo.ut.ee/primer3-0.4.0/). Gene expression was quantified by the 
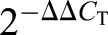
 method. Data were normalized by the expression of the *Gapdh* housekeeping gene. Statistical significance was determined by Student's *t*-test, with *P*<0.1. The list of primer sequences is displayed in Table S5.

## Supplementary Material

Supplementary information
