# Supplementary material for: Temporal analysis of hippocampal CA3 gene coexpression networks in a rat model of febrile seizures
Source: Dis Model Mech. 2018 Jan 1;11(1):dmm029074. doi: 10.1242/dmm.029074 (PMC5818071; doi:10.1242/dmm.029074)
Supplement: Supplementary information [file dmm-11-029074-s1.pdf]

Table S1. Functional enrichment analysis in modules that gain or lose connectivity after hyperthermic seizures at P12.

MODULE (GENES)	MODULE TYPE	HUBS	MAIN ENRICHED FUNCTIONS
**Blue (367)**	**Gain of connectivity**	Bcar1, Slc25a5, Entpd2, Zfp148, Gimap9, Srp72, Rarres1, Hspa9, Rassf2, Romo1	Cell cycle, Endocytosis, Focal Adhesion, Regulation of Actin Cytoskeleton, EGFR Pathway, Apoptosis, Cellular response to stress, Autophagy, DNA replication, axonogenesis, neuron cell-cell adhesion
**Green (215)**	**Gain of connectivity**	Abcb6, Exosc5, Rbm7, C1ql1, Uri1, Pycr2, Mpdz, Hdgfrp3, Cstf2, Nudt16l1	Endocannabinoid signaling, Proteoglycans in cancer, PI3K-Akt pathway, GABAergic synapse, Focal Adhesion, Class I MHC mediated antigen processing, EPH-ephrin mediated repulsion of cells, oxidative phosphorylation, cell projection organization, positive regulation of neuron differentiation
**Magenta (160)**	**Gain of connectivity**	Slc4a2, Zfp407, Nes, Etv3, Cd6, Glrx2, Phb, Clrn2, Ccnf, Kcnk2	Proteoglycans, FoxO pathway, MAPK pathway, Chromatin organization, Gastrin-CREB signaling pathway via PKC and MAPK, Regulation of synaptic transmission, regulation of membrane potential
**Salmon (132)**	**Gain of connectivity**	Cct5, Col1a2, Mien1, Mettl2b, Mxi1, Foxe1, Cfl1, Rreb1, Hyal3, Cdhr1	Oxidative phosphorylation, TGF-β Pathway, Cell cycle, NF-κB activation, Ion channel transport, response to IL-1, Wnt pathway, apoptosis, actin cytoskeleton organization, stem cell maintenance
**Darkred (97)**	**Less preserved**	Nefl, Ssbp3, Epm2aip1, Sat1, Psmg4, Cmas, Srgap3, Zc3h18, Ctss, Scml4	p53 pathway, HIF-1 pathway, Neurotrophin pathway, Wnt pathway, Apoptosis, Activation of NMDA receptor, Hemostasis, Signaling by FGFR, Cell differentiation, axonogenesis
**Royalblue (98)**	**Less preserved**	Tmem212, Tbkbp1, Nxph1, Adcyap1, Fcgr1a, Tspyl4, Eif4ebp1, Aars, Mal2, Apex1	PI3K-Akt-mTOR-signaling, EPH-Ephrin signaling, Axon guidance, SLC-mediated transmembrane transport, Signaling by VEGF, negative regulation of neuron death, neuron projection development, neurogenesis
**Grey60 (106)**	**Less preserved**	Ddx39b, Ap2s1, Ddx24, Atg16l1, Commd4, Acin1, Bloc1s2, Eif3e, Tpm3, Chmp4b	Calcium Regulation, Integrin-mediated cell adhesion, Focal Adhesion, PI3K-Akt-mTOR-signaling, Signaling by EGFR, vesicle-mediated transport, cellular response to stress, regulation of vasculature development
**Darkgreen (97)**	**Less preserved**	Prpf40a, Sort1, Cd33, Uqcrc1, Smarca4, Ubc, Rnf187, Gdi1, Cript, Otx1	Cholinergic synapse, potassium channels, Signaling by Wnt, mitotic cell cycle phase transition, T cell activation, neuron differentiation

Disease Models & Mechanisms • Supplementary information

Table S2. Functional enrichment analysis in modules that gain or lose connectivity after hyperthermic seizures at P30.

MODULE (GENES)	MODULE TYPE	HUBS	MAIN ENRICHED FUNCTIONS
**Blue (345)**	**Gain of connectivity**	Slc30a5, Hypk, Ankrd33b, Axin1, Arv1, Inip, Dscr3, Ptp4a3, Zfyve1, Acy3	Protein processing in endoplasmic reticulum, Hippo pathway, Wnt pathway, apoptosis, Chemokine receptors bind chemokines, L1CAM interactions, myeloid cell differentiation, actin filament organization, regulation of neuron apoptotic process
**Brown (301)**	**Gain of connectivity**	Osm, B2m, Wbp4, Spata18, Retsat, Pomt1, Olr546, Stra6, Pcdhga7, Usp5	Parkinson's disease, Dopaminergic and cholinergic synapses, long term potentiation, TCA cycle, cellular metabolism, proteasome, autophagy, negative regulation of apoptosis, nervous system development, negative regulation of cell differentiation
**Green (282)**	**Gain of connectivity**	Chac2, Tmed7, Ivd, Tmx3, Smarcb1, Gmpr, Sav1, Ndufb2, Cpt1a, Sqstm1	Glutamatergic synapse, GABAergic synapse, Ubiquitin-mediated proteolysis, Metabolic pathways, Oncostatin M Pathway, DNA damage response, Class I MHC mediated antigen processing, cell junction assembly, neuron projection development, synaptic transmission
**Lightcyan (165)**	**Gain of connectivity**	Tmem171, R3hdml, Chd5, Pth2, Eif1, Foxp3, Gja8, Rap2a, Polr1b, Pik3r5	Electron Transport Chain, Glutathione conjugation, DNA Repair, regulation of neuron differentiation, neurogenesis, calcium ion homeostasis
**Magenta (202)**	**Gain of connectivity**	Sapcd1, RalA, Olr567, Gpr137, Lace1, Creb3, Rabggta, Mum1, Med25	Phospholipase D signaling pathway, Actin cytoskeleton organization, Gastrin-CREB signaling pathway, positive regulation of cell adhesion and chemotaxis, stem cell maintenance, positive regulation of neurogenesis, stem cell differentiation
**Red (228)**	**Gain of connectivity**	Tmem246, Sult4a1, Vps11, Psmd8, Armc9, Dopey2, Prkar1b, Dctn2, Ankrd46, Pcyox1	Synaptic vesicle, lysosome, Leukocyte transendothelial migration, glutamatergic synapse, Regulation of Actin Cytoskeleton, Neuronal System, MHC class II antigen presentation, signaling by VEGF, signaling by EGFR, synaptic transmission, neuron projection development, neuron apoptosis, neuron differentiation
**Darkgreen (137)**	**Less preserved**	Chac1, Pif1, Hpcal4, Noxa1, Defb24, Epha10, Atp6ap1l, Zfp516, Htr5b, Ncf1	Axon guidance, Ras pathway, Long-term potentiation, Apoptosis, BDNF signaling pathway, IL-1 pathway, Cell Adhesion, EPH-Ephrin signaling, Innate immune pathway, Rho GTPase Effectors
**Pink (207)**	**Less preserved**	Sectm1b, Ube3d, Fam134b, Idh3g, Anapc13, S100b, Cpne8, Pgam1, Dpysl2, Itgb3bp	Synaptic transmission, glycolysis, oxidative phosphorylation, metabolism of amino acids, immune system, iron ion homeostasis, neuron migration, organic acid transport
**Tan (191)**	**Less preserved**	Wfdc10, Gpd1l, Sfrp5, Baiap2, Myo5b, Tmem229b, Bsdc1, Nup62, Thop1, Cacna1e	Wnt signaling pathway, MAPK pathway, Calcium signaling, Axon guidance, Neural Crest Differentiation, neuronal System, synaptic transmission, regulation of ion membrane transport, actin cytoskeleton organization, axon guidance

Table S3. Functional enrichment analysis in modules that gain or lose connectivity after hyperthermic seizures at P60.

MODULE (GENES)	MODULE TYPE	HUBS	MAIN ENRICHED FUNCTIONS
**Brown (249)**	**Gain of connectivity and Less preserved**	Rmnd5b, Bop1, Actn1, Sec14l5, Tmem25, Fkbp8, Acap3, Cacnb3, Cnrip1, Il16	Focal adhesion, stem cell pluripotency, metabolism of lipids, positive regulation of lymphocyte proliferation, negative regulation of cell migration, neuron projection development
**Midnightblue (160)**	**Gain of connectivity**	Anxa7, Snd1, Klhdc3, C5ar1, Gosr2, Bpifb6, Or10ad1, Rpp30, Rps19, Atp6v0a1	Synaptic vesicle, Glutamatergic synapse, BDNF signaling pathway, Neuronal System, Antigen processing: Ubiquitination & Proteasome degradation, Ion channel transport, synaptic transmission, neuronal plasticity
**Red (212)**	**Gain of connectivity**	Dctn1, Dapk3, Dgat1, Mus81, Unc45a, Rnft2, Dcaf15, Pomgnt2, Abcb8, Ogdh	Hippo pathway, interleukin signaling, neural crest differentiation, Extracellular matrix organization, hippocampus development, innate immune response
**Turquoise (438)**	**Gain of connectivity**	Tpd52l1, Scx, Mx1, Ednra, Ano3, Dleu7, Slc7a3, Nr2f2, Epha5, Gpr155	Calcium ion transport, cholinergic synapse, axon guidance, Wnt pathway, inflammatory regulation of TRP channels, Neuronal system, gastrin-CREB signaling pathway, Signaling by Robo, Potassium Channels, neuron migration, synaptic transmission
**Yellow (235)**	**Gain of connectivity**	Bckdk, Rmdn1, Ninj1, Robo1, Tst, Dchs1, Galt, Lsm3, Olr733, Gja10	Metabolic pathways, neuroactive ligand-receptor interaction, GABAergic synapse, Extracellular matrix organization, neuron projection development, synaptic transmission, neuron differentiation, neurogenesis
**Greenyellow (176)**	**Less preserved**	Ptprt, Armcx1, Tpcn1, Ube2m, Acbd5, Mta2, Celf4, Taok2, Pomgnt1, Slc16a13	Wnt pathway, p53 pathway, Axonal guidance, regulation of RNA splicing, neuron cell morphogenesis, regulation of protein ubiquitination, synaptic transmission
**Orange (114)**	**Less preserved**	Olr1516, March9, Ache, Abcc6, Dusp9, Dynlt1, Sin3b, Sept2, Rnf182, Gltp	Long-term potentiation, cholinergic synapse, axon guidance, Kit receptor signaling pathway, signaling by EGF, cell junction assembly, cell differentiation, positive regulation of cell migration
**Skyblue (97)**	**Less preserved**	Calr, Sobp, Acin1, Rabgap1, Nrgn, Epn1, Sstr4, Ring1, Scaf1, Sncb	Endocytosis, Notch Pathway, Sphingolipid metabolism, ERBB signaling, nervous system development, small GTPase mediated signal transduction

Disease Models & Mechanisms • Supplementary information

Table S4. Functional enrichment analysis in modules that gain or lose connectivity after hyperthermic seizures at P120.

MODULE (GENES)	MODULE TYPE	HUBS	MAIN ENRICHED FUNCTIONS
**Brown (249)**	**Gain of connectivity**	Dgkz, Armt1, Acp2, Robo2, Gria1, Ppp2r5c, Pard6a, Mast3, Scd2, Pdpk1	Synaptic transmission, Long-term potentiation, Leukocyte transendothelial migration, Focal adhesion, Regulation of actin cytoskeleton, Rap1 pathway, Axon guidance, signaling by Wnt, Signaling by Robo, neuron projection development, neuron differentiation
**Blue (264)**	**Gain of connectivity**	Pdap1, Pcbd2, Eif3f, Npdc1, Rnf34, Ndufb3, Shank3, Ndufs5, Bmpr1b, Map3k7	MAPK signaling pathway, Extracellular matrix organization, regulation of synaptic transmission, apoptosis, regulation of cell morphogenesis involved in differentiation, negative regulation of neurogenesis, neuron projection guidance, response to oxidative stress
**Pink (208)**	**Gain of connectivity**	Cerk, Prpf8, Ipo13, Ncaph2, Sptan1, Apc2, Exosc7, Arhgdia, Exoc6b, Kdm3a	Alzheimer's disease, Complement and coagulation cascades, Foxo signaling pathway, Phospholipase D signaling pathway, Toll-like receptor signaling pathway, regulation of actin cytoskeleton, Antigen processing, Wnt signaling pathway
**Grey60 (128)**	**Less preserved**	Ciz1, Vps4b, Cryga, Gatad2a, Tnrc6a, Cybrd1, Med1, Casp7, Abcd4, Sbno2	Fc gamma R-mediated phagocytosis, positive regulation of lymphocyte proliferation, positive regulation of lymphocyte activation, regulation of I-kappaB kinase/NF-kappaB signaling, cell projection organization, regulation of cell morphogenesis
**Skyblue (93)**	**Less preserved**	Polr1c, Ska3, Hyal2, Hmmr, Ttc21b, Avl9, Hacd2, Mecr, Vom1r81, Prrc1	TNF-alpha NF-kB Pathway, Hyaluronic metabolism, regulation of Wnt pathway, negative regulation of neuron differentiation, actin filament organization, negative regulation of cell migration, regulation of MAPK activity
**Lightgreen (125)**	**Less preserved**	Tbx3, Ciapin1, Adprm, Scly, Atf4, Commd8, Sf3b3, Bpgm, Ccdc50, Stat1	Metabolism, Extracellular matrix organization, regulation of anion transport, neuron projection development

Disease Models & Mechanisms • Supplementary information

Table S5. List of primer sequences used for the quantitative real time polymerase chain reactions.

Gene	Primer Code	Sequence (5' to 3')
**PTGIR**	PTGIR FW	TGGGACGATGCTGTGTGA
	PTGIR RV	GAAAGCGTAGATGGAAGGCAA
**SOX9**	SOX9 FW	AGGAAGCTGGCAGACCAGTA
	SOX9 RV	ACGAAGGGTCTCTTCTCGCT
**TRIP12**	TRIP12 FW	CCAACCCAGAAATCAACCAGTC
	TRIP12 RV	GATTTCCAACATGGCCCGGGAG
**RHOX8**	RHOX8 FW	TGCCTGGACCCCTACTATTG
	RHOX8 RV	CTGGCTGGCACATAGTCCTG
**GAPDH**	GAPDH FW	GACATGCCGCCTGGAGAAAC
	GAPDH RV	AGCCCAGGATGCCCTTTAGT

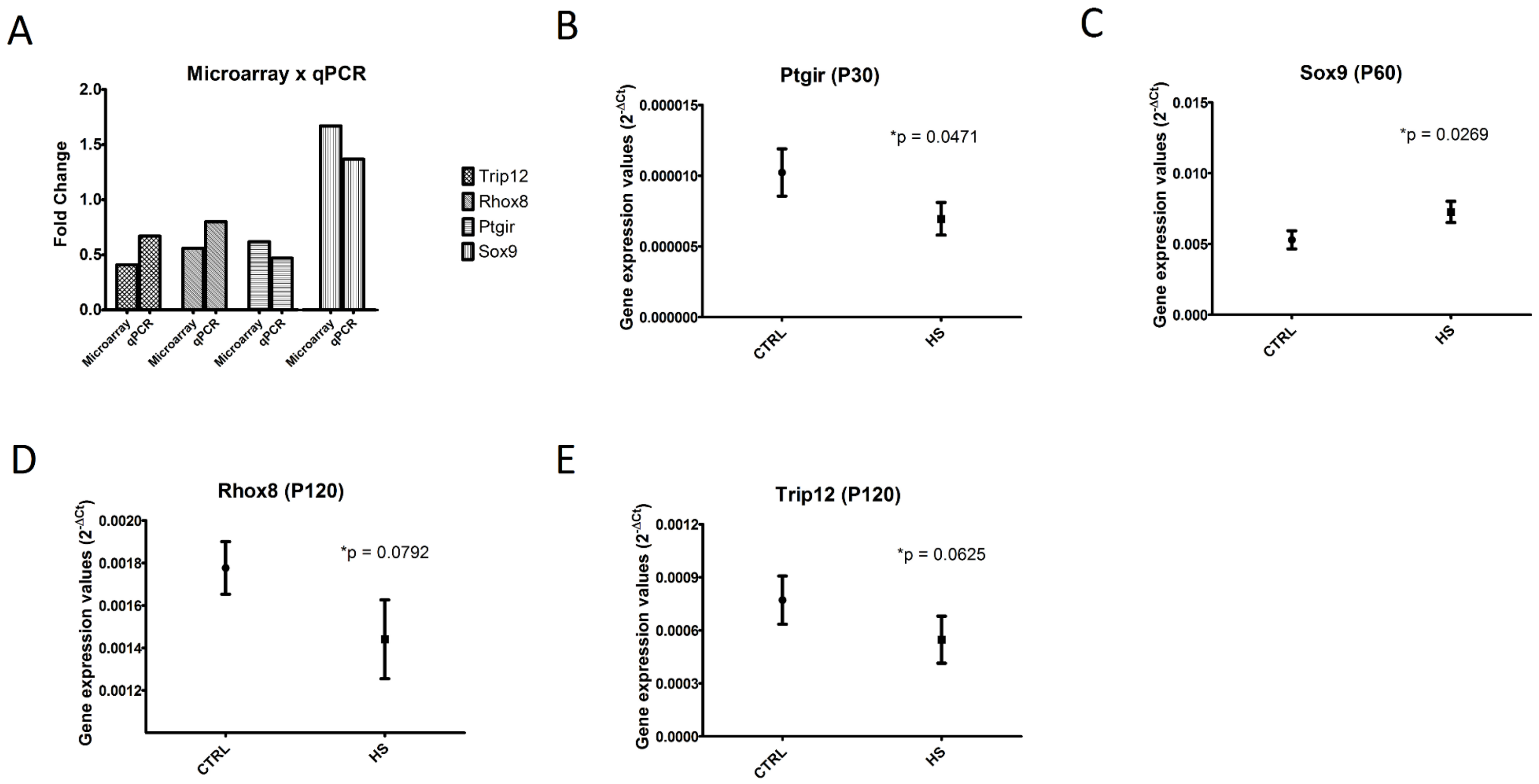

Figure S1. Comparison of microarray and qPCR results for selected genes. The genes Trip12, Rhox8, Ptgir and Sox9 were selected for validating microarray results using qPCR analysis.

Disease Models & Mechanisms • Supplementary information

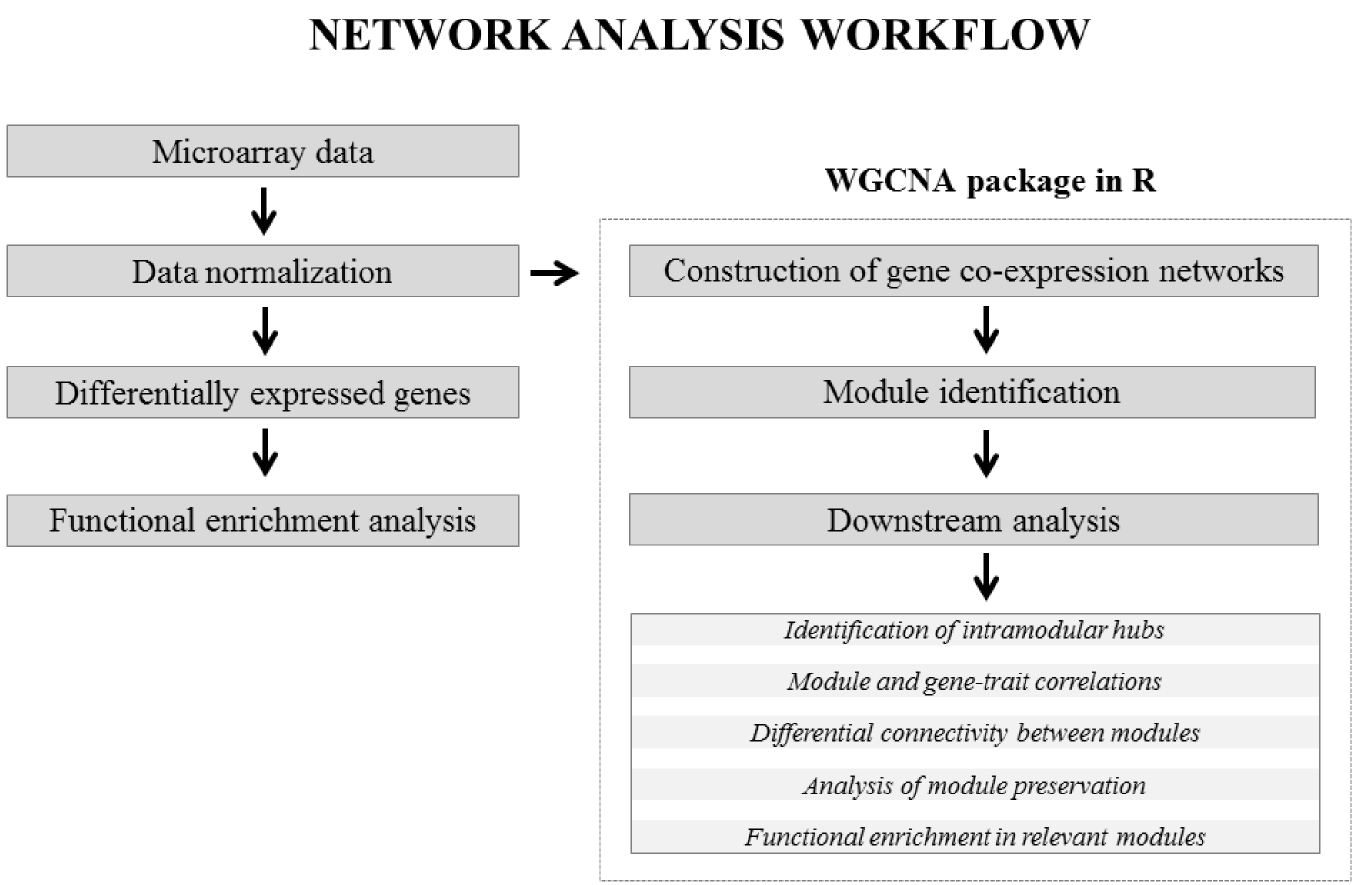

Figure S2. Schematic representation of the network analysis workflow. Microarray data was log2-transformed and normalized using lowess normalization. This data was used for weighted correlation network analysis. The network analysis consisted in building gene co-expression networks, identifying network modules and hubs, performing gene-trait correlations and examining module preservation and connectivity changes. Relevant modules were also functionally enriched to identify functions associated to hyperthermic seizures.

Disease Models & Mechanisms • Supplementary information
